# HLA Diversity in Saudi Population: High Frequency of Homozygous HLA Alleles and Haplotypes

**DOI:** 10.3389/fgene.2022.898235

**Published:** 2022-06-09

**Authors:** Aziz Alami Chentoufi, F. Aytül Uyar, Hiba A. Chentoufi, Khalid Alzahrani, Maria Paz, Ahmed Bahnassy, Ghaleb Elyamany, Assem Elghazaly

**Affiliations:** ^1^ King Fahad Medical City, Riyadh, Saudi Arabia; ^2^ Department of Medical Microbiology, Faculty of Health Sciences, University of Pretoria, Pretoria, South Africa; ^3^ Department of Physiology, Istanbul Medical Faculty, Istanbul University, Istanbul, Turkey; ^4^ Biomedical Sciences, University of Ottawa, Ottawa, ON, Canada; ^5^ Department of Central Military Laboratory and Blood Bank, Prince Sultan Military Medical City, Riyadh, Saudi Arabia; ^6^ Department of Hematology/Oncology, King Saud Medical City, Riyadh, Saudi Arabia

**Keywords:** HLA, genetic diversity, allele frequency, allele and haplotype homozygosity, TA-GVHD

## Abstract

Human leukocyte antigens (HLA) diversity has a tremendous impact on shaping the transplantation practices, transfusion-associated graft versus host disease prevention strategies, and host–pathogen interactions. Here, we conducted a retrospective study of HLA class I and class II homozygosity at allelic and haplotype levels in unrelated individuals genotyped from 2012 to 2016 in a tertiary hospital in the capital of Saudi Arabia. Among 5,000 individuals, 2,773 individuals meet inclusion criteria and were retrospectively analyzed for HLA-A, -B, -C–DRB1, and -DQB1 homozygosity at allelic and haplotype levels. HLA molecular typing was performed using a commercial reverse sequence-specific oligonucleotide (rSSO) kit. We were able to identify 15 HLA-A, 20 HLA-B, 11 HLA-C, 13 HLA-DRB1, and five HLA-DQB1 homozygous alleles demonstrating a very low genetic diversity in the Saudi population. The highest homozygosity in HLA class I was found in locus C followed by A and B (20.3% > 16.1% > 15.5%; *p* < 0.001) where the most homozygote alleles were A*02 (9.2%), B*51 and B*50 (5.7% and 3.7%), and C*07, C*06, and C*15 (7.2%, 5.48%, and 3.3%) and in HLA class II, the highest homozygosity was found in locus DQB1 compared to DRB1 (31.71% > 19.2%; *p* < 0.001), with the most common homozygote alleles being DRB1*07 and DRB1*04 (5.33% and 4.2%) and DQB1*02, DQB1*06, and DQB1*03 (13.55%, 7.92%, and 7.64%). The frequency of finding an individual with one homozygote allele was (24.6%), two homozygote alleles (13.5%), three homozygote alleles (4.7%), four homozygote alleles (3.4%), and five alleles were (4.8%). The most frequent homozygote haplotypes are A*23∼C*06∼B*50∼DRB1*07∼DQB1*02 and A*02∼C*06∼B*50∼DRB1*07∼DQB1*02. This study shows low diversity of both class I and II alleles and haplotypes in the Saudi population, which would have a significant impact on shaping the transplantation practices, transfusion-associated graft versus host disease prevention strategies, and host–pathogen interactions.

## Introduction

Human leukocyte antigens (HLA) molecules, also known as MHC molecules (major histocompatibility complex), are encoded by the most polymorphic cluster of genes in the human genome located on the short arm of chromosome 6 (Marrack and Kappler, 1986). Classically divided into HLA class I (HLA-A, -B, and -C) and HLA class II (HLA-DR, -DQ, and -DP) with more than 27.000 HLA alleles sequences (Robinson et al., 2020) encoding for molecules critical to immunity, including antigen processing and presentation to immune effector CD4^+^ and CD8^+^ T-cells in an HLA-restricted manner, as such, HLA molecules have been extensively studied in the context of organ and hematopoietic cell transplantation and infectious and autoimmune disease associations (Shiina et al., 2004; Dehn et al., 2008; Trowsdale and Knight, 2013; Dendrou et al., 2018).

Up to date, HLA class I and class II alleles and haplotype frequencies were characterized in different populations around the globe including the Saudi population (Maiers et al., 2007; Saw et al., 2015; Creary et al., 2019; Jawdat et al., 2020). Understanding HLA diversity in these populations has a tremendous impact on clinical outcomes of solid organ and hematopoietic cell transplantation where a mismatch in HLA class I and/or class II alleles between donors and recipients represent a major cause for graft failure, rejection, and graft versus host disease (GVHD) (Lee et al., 2007; Dehn et al., 2008; Petersdorf, 2008; Kunze-Schumacher et al., 2014). In addition, HLA diversity presents an important challenge in transfusing medicine where HLA homozygosity represents the major cause of transfusion-associated-graft versus host disease (TA-GVHD) in immunocompetent patients. TA-GvHD is a very rare but fatal complication that occurs following transfusion of lymphocyte-containing blood components such as packed red blood cells and platelets (Treleaven et al., 2011).

The HLA genetic diversity has been shown to play a critical role in developing an effective host response against pathogens, disease progression, and outcome. Indeed, it has been shown that HLA heterozygous individuals have an advantage over homozygous individuals in presenting pathogen-derived epitopes to effector CD8^+^ and CD4^+^ T-cells (Doherty and Zinkernagel, 1975; Thursz et al., 1997; Penn et al., 2002). Multiple infectious disease studies have confirmed the negative clinical impact of HLA homozygosity on disease development and outcome (Thursz et al., 1997; Carrington et al., 1999; Burgner et al., 2006). Similarly, it has been shown for non-infectious neoplasms that HLA homozygosity reduces the ability of the immune system to differentiate cancer cells from noncancer cells (Hojjat-Farsangi et al., 2008; Shah et al., 2011).

The Kingdom of Saudi Arabia (KSA) constitutes the majority of the Arab Peninsula in southwest Asia, with a land area of about 2,000,000 km^2^. According to the 2020 survey, the KSA population is 35 million with an annual growth rate of 2.4. The gender ratio of the Saudi population is 1.04 (General Authority of Statistics, 2016). At least 24.8% of the whole population is less than 15 years and people in the working age who range between 15–64 years old represent 72%. The Saudi Population consists of a multi-ethnic population, of which the largest portion (90% of which) constitutes Arab. KSA is characterized by a large migrant population, which forms 36.3% of its residents, the majority of whom are of Asian and African ethnicities. 26.3% of immigrants are native to India (General Authority of Statistics, 2016). Most of the marriage is between Saudi people, and the overall rate of consanguinity is about 57.7%, one of the highest worldwide (El-Hazmi et al., 1995), a trend that did not show a changeover generation (Warsy et al., 2014).

The aim of this work is to study the prevalence of homozygosity of HLA class I and Class II alleles and haplotypes which would reflect the degree of genetic diversity (or similarity) in the Saudi population. This study would pave the way to better transplantation practices, transfusion-associated graft versus host disease prevention strategies, and host–pathogen interactions in the Saudi population.

## Materials and Methods

### Cohort Study

This is a retrospective study, using already available HLA typing results of Saudi individuals (HSCT-unrelated donors). The study was performed on 5,000 individuals, who have been HLA-typed for HLA-class I (A, B, and C) and HLA-class II (DR and DQ) in the HLA laboratory at KFMC between January 2012 and June 2016. Only 2,773 unrelated individuals were included in this cohort study representing Saudi Arab ethnicity. Saudi ethnicity was defined as Arabs belonging to known Saudi tribes and citizens of the kingdom of Saudi Arabia. The study population shows that they were in the age range of <25 years: 15%; 25–45 years: 55%; and 45–76 years: 30%. The male: female ratio of patients was 51%:49%. The retrospective study was approved for human investigations at King Fahad Medical Hospital Tertiary Hospital under an institutional review board–approved protocol (IRB# 15-198).

### Blood Samples, DNA Extraction, and Quantification

Blood samples were drawn in the ward in 2-ml EDTA tubes and sent to the HLA laboratory for HLA typing at KFMC. For DNA extraction from blood samples, an automated MagNA Pure Compact System (Roche Life Sciences, Indianapolis, United States) was used. The protocol was performed as per the manufacturer’s instructions without modifications. DNA extraction: The extracted DNA was quantified using Nanodrop 2000 C (Thermo Scientific, Wilmington, DE, United States) and normalized to the desired concentration as recommended by the manufacturer’s protocol.

### HLA-A, -B, -C, -DRB1, and -DQB1 Genotyping Using rSSO

HLA-A typing was performed using a commercial reverse sequence-specific oligonucleotides kit (SSO; One Lambda, Canoga Park, CA). Briefly, extracted and quantified genomic DNA from the blood has been subjected to a PCR reaction using primers specific for areas of the exon 2 and 3 for HLA class I (HLA-A, B, and C) and the exon 2 for HLA-DRB1 and HLA-DQB1. The PCR product was hybridized with a mixture of beads (*n* = 100), each one having a specific sequence and color. Then, the beads were acquired on the Luminex platform and analyzed by HLA Fusion Software (One Lambda, Inc., Canoga Park, CA). To make sure that the obtained homozygosity is not false results commonly obtained with rSSO, we confirmed our results by family segregation study and/or sequence-based typing for the concerned homozygous allele.

### Statistical Analysis

The HLA class I and class II allele homozygosity frequencies were calculated by dividing the number of homozygote individuals for this allele by the total number of homozygote individuals at that locus. Allele homozygosity population frequency was calculated by dividing the number of homozygote individuals for this allele by the total number of studied individuals. All frequencies were calculated from an Excel spreadsheet counts and using Fisher’s exact test (two-tailed) to calculate the *p* value (*p* value < 0.05 is considered significant).

The population analyses and genetic diversity measures were calculated using Arlequin 3.5 software. Allele frequencies and five locus haplotype frequencies were determined by the expectation-maximization (EM) algorithm. For testing the deviation from Hardy Weinberg equilibrium, a Markov chain algorithm was performed (Excoffier and Lischer, 2010). Alleles are said to be in Hardy Weinberg equilibrium when the observed heterozygote frequencies do not significantly differ from the expected frequencies (*p* > 0.05).

## Results

### HLA Class I Allele Frequency Distribution of Homozygosity

**FIGURE 1:**
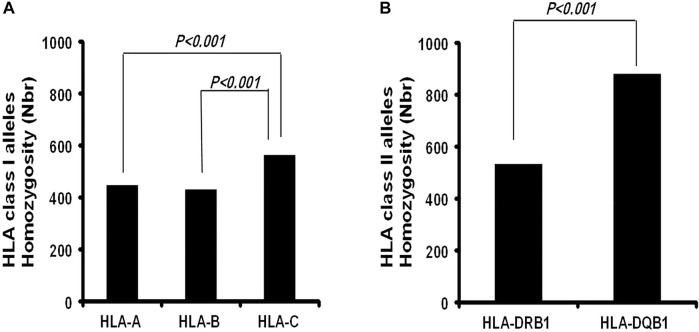
Comparison of the Frequency of homozygosity between HLA class I Loci and HLA class II loci in Saudi population.

In our cohort, 15 HLA-A, 20 HLA-B, and 11 HLA-C homozygous alleles were identified (Tables 1–3). Of the 15 HLA-A homozygous alleles identified, only one with the frequency of homozygosity above 5% was found (Table 1). This allele was HLA-A*02 (9.2%). Similarly, of the 20 HLA-B homozygous alleles identified in this population, only one allele B*51 (5.7%) showed a frequency of homozygosity above 5% (Table 2). Among the 11 HLA-C homozygous alleles identified in this population, two alleles C*07 and C*06 (7.2% and 5.48%) showed a frequency of homozygosity above 5% (Table 3). Comparatively, as shown in (Figure 1A), HLA-C showed the highest frequency of homozygosity followed by HLA-A and HLA- B (20.3% > 16.1% > 15.5%; *p* < 0.001). In order to analyze the influence of gender on HLA allele frequencies, we have calculated HLA class I and class II allele frequencies in males and females. A comparison between male and female allele frequencies of HLA-A, -B, -C, - DRB1, and DQB1 loci show that there were no significant differences between male and female in term of allele frequencies, demonstrating that gender has a nonsignificant influence on HLA allele frequencies in Saudi population (Supplementary Table S1).

**TABLE 1:** List of HLA-A alleles detected in locus A, the frequency of homozygosity, and allele frequencies.

HLA-A alleles	Number of homozygous individuals	Homozygosity allele frequency (%)	Homozygosity population frequency (%)	Allele frequency (%)
**01**	**28**	**6.25**	**1.0**	**7.30**
**02**	**255**	**56.9**	**9.2**	**28.38**
03	13	2.9	0.5	6.40
11	5	1.1	0.2	2.85
23	26	5.8	0.9	6.15
**24**	**32**	**7.1**	**1.2**	**7.72**
26	20	4.5	0.7	5.84
29	8	1.8	0.3	2.78
30	22	4.9	0.8	6.11
**31**	**27**	**6.0**	**1.0**	**7.10**
33	8	1.8	0.3	4.06
34	1	0.2	0.04	0.45
66	1	0.2	0.04	0.49
68	1	0.2	0.04	9.63
74	1	0.2	0.04	0.67
Total	**448**	**100**	**16.1**	**95.93**

Bold values indicate the highest frequencies.

**TABLE 2:** List of HLA-B alleles detected in locus B and the frequency of homozygosity and allele frequencies.

HLA-B alleles	Number of homozygous individuals	Homozygosity allele frequency (%)	Homozygosity population frequency (%)	Allele frequency (%)
04	1	0.2	0.04	0.04
**07**	**36**	**8.4**	**1.3**	**9.02**
08	25	5.8	0.9	8.62
13	3	0.7	0.1	0.86
15	25	5.8	0.9	5.97
18	2	0.5	0.1	2.45
27	3	0.7	0.1	1.55
35	20	4.6	0.7	6.00
37	4	0.9	0.1	1.03
38	3	0.7	0.1	1.15
39	8	1.9	0.3	2.57
40	6	1.4	0.2	1.71
41	7	1.6	0.3	3.42
44	3	0.7	0.1	2.05
45	3	0.7	0.1	0.83
47	1	0.2	0.04	0.31
**50**	**102**	**23.7**	**3.7**	**16.35**
**51**	**158**	**36.7**	**5.7**	**19.94**
52	1	0.2	0.04	2.20
53	8	1.9	0.3	3.73
57	1	0.2	0.04	1.01
58	10	2.3	0.4	2.94
73	1	0.2	0.04	0.85
Total	**431**	**100**	**15.5**	**94.6**

Bold values indicate the highest frequencies.

**TABLE 3:** List of HLA-C alleles detected in locus C and the frequency of homozygosity and allele frequencies.

HLA-C alleles	Number of homozygous individuals	Homozygosity allele frequency (%)	Homozygosity population frequency (%)	Allele frequency (%)
01	7	1.24	0.25	2.13
02	10	1.77	0.36	2.70
03	9	1.60	0.32	4.00
**04**	**50**	**8.87**	**1.80**	**10.13**
**06**	**152**	**26.95**	**5.48**	**19.54**
**07**	**199**	**35.28**	**7.2**	**24.97**
12	17	3.01	0.61	6.79
14	8	1.42	0.30	2.29
**15**	**92**	**16.31**	**3.3**	**15.00**
16	12	2.13	0.43	5.28
17	8	1.42	0.30	3.89
Total	**564**	**100**	**20.3**	**96.72**

Bold values indicate the highest frequencies.

### HLA Class II Allele Frequency Distribution of Homozygosity

As expected, the HLA class II loci are less diverse than the class I loci in this population. Only a total of 13 HLA-DRB1 and five HLA-DQB1 homozygous alleles (Tables 4, 5) were identified. HLA-DQB Class II homozygous alleles with frequencies above 5% in this population were HLA-DQB1*02, -DQB*06, and -DQB*03 (13.55%, 7.92%, and 7.64%) as shown in Table 4. Only one HLA-DRB1 homozygous allele has shown a frequency above the 5% in the population: HLA-DRB1*07 (5.33%) as shown in Table 5. Comparatively, as shown in Figure 1B, the highest homozygosity in HLA class II is found in locus HLA-DQB1 compared to HLA-DRB1 (31.71% > 19.2%; *p* < 0.001).

**TABLE 4:** List of HLA-DRB1 alleles detected in locus DRB1 and the frequency of homozygosity and allele frequencies.

HLA-DRB1 alleles	Number of homozygous individuals	Homozygosity allele frequency (%)	Homozygosity population frequency (%)	Allele frequency (%)
01	10	1.88	0.36	4.24
02	1	0.19	0.04	0.11
**03**	**75**	**14.07**	**2.70**	**15.02**
**04**	**116**	**21.76**	**4.2**	**16.91**
05	1	0.19	0.04	0.14
**07**	**148**	**27.77**	**5.33**	**19.34**
08	6	1.13	0.22	1.73
10	14	2.63	0.50	3.68
11	18	3.38	0.65	8.38
**13**	**75**	**14.07**	**2.70**	**14.75**
14	5	0.94	0.18	1.28
**15**	**52**	**9.76**	**1.88**	**10.98**
16	12	2.25	0.43	2.65
Total	**533**	**100**	**19.2**	**99.21**

Bold values indicate the highest frequencies.

**TABLE 5:** List of HLA-DQB1 alleles detected in locus DQB1 and the frequency of homozygosity and allele frequencies.

HLA-DQB1 alleles	Number of homozygous individuals	Homozygosity allele frequency (%)	Homozygosity population frequency (%)	Allele frequency (%)
01	1	0.1	0.036	0.11
**02**	**376**	**42.7**	**13.55**	**34.23**
**03**	**212**	**24.1**	**7.64**	**25.42**
**05**	**71**	**8.1**	**2.56**	**13.35**
**06**	**220**	**25**	**7.92**	**25.11**
Total	**880**	**100**	**31.71**	**98.22**

Bold values indicate the highest frequencies.

For each HLA locus, we observed a statistically reduced heterozygosity compared with the expected heterozygosity as shown by Hardy Weinberg equilibrium analysis (Supplementary Table S2). As displayed in Supplementary Table S2, the HWE deviation was observed for the heterozygote component of the HLA-A, -B, -C, -DRB1, and -DQB1 loci data, suggesting that this population may have been positively selected. Excess homozygosity observed in this study is similar to what has been reported previously by Hajeer et al. (2013) and Jawdat et al. (2012).

### Class I and Class II Loci and Haplotypes Homozygosity

The frequency of allele homozygosity was overall lower in HLA class I genes than that in class II genes. Among the 2,773 individuals who were fully typed at class I and class II loci, 683 individuals were homozygous at one class I or class II allele (24.6%), 374 individuals (13.5%) were homozygous at two class I and/or HLA class II alleles, 131 individuals (4.7%) were homozygous at three class I and/or class II alleles, 94 individuals (3.4%) were homozygous at four class I and/or HLA class II alleles, and interestingly among the 2,773 individuals who were fully typed at class I and class II loci, 134 (4.8%) were homozygous at the five loci of HLA class I and class II (Table 6). The most frequent homozygote haplotypes are A*23∼C*06∼B*50∼DRB1*07∼DQB1*02 (0.8%) and A*02∼C*06∼B*50∼DRB1*07∼DQB1*02 (0.55%) (Table 7). Saudi population showed higher HLA homozygous haplotype frequency (4.8%) than other populations such as Japanese (1%) and East African (0.96) (Supplementary Table S3) (Kawashima et al., 2012; Peterson et al., 2014).

**TABLE 6:** Frequency to find one, two, three, four, or five homozygote HLA class I and/or alleles in our cohort study.

Number of homozygous alleles	Number of individuals	Homozygosity population frequency (%)
0	1,375	49.5
1	683	24.6
2	374	13.5
3	131	4.7
4	94	3.4
**5**	**134**	**4.8**
Total	**2,775**	**100**

Bold values indicate the highest frequencies.

**TABLE 7:** Frequency of HLA- A ∼ C ∼ B ∼ DRB1∼DQB1 haplotype homozygosity and haplotype frequency in Saudi population.

HLA- A ∼ C ∼ B ∼ DRB1∼DQB1 haplotypes	Homozygosity haplotype frequency (%)	Homozygosity population frequency (%)	Haplotype frequency (%)
A*23∼C*06∼B*50∼DRB1*07∼DQB1*02	18.33	0.8	3.35
A*02∼C*06∼B*50∼DRB1*07∼DQB1*02	12.3	0.55	4.64
A*26∼C*07∼B*08∼DRB1*03∼DQB1*02	4.1	0.2	1.77
A*02∼C*15∼B*51∼DRB1*04∼DQB1*03	3.28	0.15	2.80
A*02∼C*07∼B*07∼DRB1*03∼DQB1*02	3.28	0.15	0.76
A*31∼C*15∼B*51∼DRB1*13∼DQB1*06	2.46	0.1	1.50
A*01∼C*17∼B*41∼DRB1*07∼DQB1*03	2.45	0.1	0.81
A*24∼C*04∼B*35∼DRB1*15∼DQB1*06	2.45	0.1	0.37
A*24∼C*07∼B*08∼DRB1*03∼DQB1*02	1.63	0.07	1.15

## Discussion

In this study, homozygosity at the HLA class I and class II loci was significantly higher than that in any other population. This low frequency of diversity in the Saudi population would have a significant impact in shaping the transplantation practices, transfusion-associated graft versus host disease prevention strategies, host–pathogen interaction, and management of recessive mutation expression in the population. HLA class I and II allele frequency distribution in the Saudi population is of prominence in disease association studies, transfusion medicine practices, and vaccine development (Ollier et al., 1985; Sheth et al., 1985; Jawdat et al., 2009; Al-Awwami et al., 2012; Jawdat et al., 2012; Hajeer et al., 2013; Osman et al., 2014). While many genetic studies of the diversity of different populations have been conducted, very few have analyzed HLA class I and class II allele homozygosity (Gladman et al., 1986; Rohowsky-Kochan et al., 1998; Azurdia et al., 1999).

We found here that the Saudi population presents high levels of homozygosity at allele and haplotype levels. The most HLA class I homozygote alleles (>5%) were HLA-A*02, HLA-B*51, and HLA- C*07 and -C*06. HLA-C showed the highest frequency of homozygosity followed by HLA-A and HLA- B (20.3% > 16.1% > 15.5%; *p* < 0.001). In HLA class II, the most frequent homozygotes alleles were HLA-DRB1*07 and HLA-DQB1*02 and -DQ*06, and -DQ*03. The highest homozygosity in HLA class II is found in locus DQB1 compared to DRB1* (31.71% > 19.2%; *p* < 0.001). This alleles homozygosity impacted the levels of haplotype homozygosity, with the most homozygous haplotypes in Saudi population being A*23∼C*06∼B*50∼DRB1*07∼DQB1*02 (0.8%) and A*02∼C*06∼B*50∼DRB1*07∼DQB1*02 (0.55%), which are among the most frequent haplotypes in the Saudi population and the Arab population at large (Jawdat et al., 2020; Hajjej et al., 2018; Askar et al., 2013). Hardy Weinberg equilibrium analysis shows an excess of homozygotes in this cohort (Supplementary Table S2). This phenomenon has been previously described (Kanda et al., 2016; Jawdat et al., 2019), which might be explained by the high level of consanguineous marriages among the Saudi population (El-Hazmi et al., 1995).

HLA homozygosity has many advantages in allogeneic bone marrow transplantation such as the limited number of alleles to match and has been shown to give comparable results to haploidentical transplant (Anasetti et al., 1990; Morishima et al., 2020; Fakhoury et al., 2021). In a hematopoietic stem cell transplantation setup, an HLA matching sibling is an ideal donor because of the low risk of GVHD and graft rejection. The incidence of GVHD is proportional to the degree of HLA mismatch (Kanda et al., 2015). The mismatch is in the graft-versus-host direction when a mismatched allele of the donor is homozygous. A mismatched antigen in the GVH direction can be a major target for donor T cells and can cause GVHD, while the mismatch is in the host-versus-graft direction when a mismatched allele of the recipient is homozygous. An antigen in the HVG direction can be a major target for the remaining recipient T cells and can lead to graft rejection (Arima et al., 2018). Interestingly, a study from Japan showed a reduced risk of relapse after HLA matched transplantation in homozygous HLA-C1 patients with acute myeloid leukemia and chronic myeloid leukemia but not with acute lymphoblastic leukemia (Ito et al., 1988).

For these patients with HLA homozygous haplotype, related family donors who are haploidentical with no HLA mismatch in the GVHD direction are willingly available for hematopoietic cell transplantation (HCT). Kanda et al. have recently shown that patients with HLA homozygous haplotypes achieved better transplantation outcomes when the donor is an HLA-matched sibling than with HLA heterozygous haplotypes and when it is needed, transplantation from a haploidentical donor without T cell depletion is a viable option, given the comparable transplant outcomes for heterozygous-to-homozygous HCT and HLA-matched sibling heterozygous HCT (Morishima et al., 2020), suggesting that the Saudi population will have more success rate in HCT haploidentical related donor transplant.

HLA association studies with TA-GVHD have shown that homozygosity was associated with worse clinical outcomes. TA-GVHD in immunocompetent patients occurs mostly when lymphocytes are transfused from an HLA homozygous into an HLA heterozygous patient who shares one haplotype with the donor (Juji et al., 1989; Uchida et al., 1994). It was assumed that recipients are not capable of recognizing donor lymphocytes as foreign because they have similar HLA. This is exactly similar to what happens in mice when T cells are transferred from an inbred homozygous parent to their F1-hybrid offspring (Mitsunaga et al., 1992). Previously, typing HLA before and after the TA-GVHD occurs was so difficult because pancytopenia progresses so rapidly that there are not sufficient lymphocytes for HLA typing. Therefore, with the development of high-throughput HLA typing taking only a few hours using polymerase chain reaction (PCR) amplification technologies, some centers already have started using this procedure (Mitsunaga et al., 1998). The knowledge of the diversity of HLA alleles and haplotypes of the Saudi population can contribute to the understanding of how host genetic factors influence transfusion associated-graft versus host disease (TA-GvHD) risk and transfusion practice in Saudi Arabia. Such knowledge would guide decision-making regarding prophylactic blood irradiation, that is, whether to continue following the current as indicated irradiation policy or it would be safer to shift to the universal irradiation practice.

In conclusion, this work showed a high prevalence of homozygosity of HLA class I and class II alleles and haplotypes in the Saudi population, which reflects the degree of genetic diversity (or similarity) in this population. We believe that these findings would pave the way to better transplantation practices and transfusion-associated graft versus host disease prevention strategies in the Saudi population.

## Data Availability

The raw data supporting the conclusion of this article will be made available by the authors, without undue reservation.
