# Supplementary material for: HLA Diversity in Saudi Population: High Frequency of Homozygous HLA Alleles and Haplotypes
Source: Front Genet. 2022 Jun 9;13:898235. doi: 10.3389/fgene.2022.898235 (PMC9218871; doi:10.3389/fgene.2022.898235)
Supplement: Supplementary file 1 [file DataSheet1.docx]

**Table 1 Supplementary**. Comparison between male and female allele frequencies of HLA-A, -B, -C, - DRB1 and DQB1 loci in Saudi population.

| Allele | Male  (n=1341) | Female  (n=1286) |  | Allele | Male  (n=1341) | Female  (n=1286) |  | Allele | Male  (n=1341) | Female  (n=1286) |
| --- | --- | --- | --- | --- | --- | --- | --- | --- | --- | --- |
| A*01 | 0.06637 | 0.08048 |  | **B*01** | 0.00000 | 0.00039 |  | **DRB1*01** | 0.04474 | 0.04160 |
| A*11 | 0.02125 | 0.03460 |  | **B*04** | 0.00037 | 0.00039 |  | **DRB1*02** | 0.00149 | 0.00078 |
| A*02 | 0.28300 | 0.28577 |  | **B*05** | 0.00037 | 0.00000 |  | **DRB1*03** | 0.14616 | 0.15280 |
| A*23 | 0.05891 | 0.06415 |  | **B*07** | 0.08837 | 0.09292 |  | **DRB1*04** | 0.16928 | 0.16563 |
| A*24 | 0.07942 | 0.07387 |  | **B*08** | 0.08501 | 0.08981 |  | **DRB1*05** | 0.00149 | 0.00156 |
| A*26 | 0.06339 | 0.05404 |  | **B*13** | 0.00671 | 0.00933 |  | **DRB1*06** | 0.00037 | 0.00117 |
| A*29 | 0.03281 | 0.02255 |  | **B*14** | 0.01864 | 0.01555 |  | **DRB1*07** | 0.19799 | 0.18974 |
| A*03 | 0.06339 | 0.06415 |  | **B*15** | 0.05183 | 0.06765 |  | **DRB1*08** | 0.01790 | 0.01555 |
| A*30 | 0.06376 | 0.06065 |  | **B*18** | 0.02498 | 0.02488 |  | **DRB1*09** | 0.00485 | 0.00117 |
| A*31 | 0.06674 | 0.07737 |  | **B*27** | 0.01939 | 0.01322 |  | **DRB1*10** | 0.03878 | 0.03771 |
| A*32 | 0.03915 | 0.03149 |  | **B*33** | 0.00037 | 0.00000 |  | **DRB1*11** | 0.08389 | 0.08515 |
| A*33 | 0.04325 | 0.03655 |  | **B*35** | 0.05481 | 0.06337 |  | **DRB1*12** | 0.00224 | 0.00467 |
| A*34 | 0.00373 | 0.00583 |  | **B*37** | 0.00858 | 0.01283 |  | **DRB1*13** | 0.15436 | 0.13997 |
| A*36 | 0.00037 | 0.00039 |  | **B*38** | 0.01417 | 0.00933 |  | **DRB1*14** | 0.01007 | 0.01633 |
| A*38 | 0.00037 | 0.00000 |  | **B*39** | 0.02573 | 0.02527 |  | **DRB1*15** | 0.09769 | 0.12247 |
| A*43 | 0.00075 | 0.00078 |  | **B*40** | 0.01641 | 0.01905 |  | **DRB1*16** | 0.02796 | 0.02372 |
| A*58 | 0.00000 | 0.00039 |  | **B*41** | 0.03281 | 0.03538 |  | **DRB1*17** | 0.00075 | 0.00000 |
| A*06 | 0.00075 | 0.00039 |  | **B*42** | 0.00746 | 0.00855 |  |  |  |  |
| A*66 | 0.00634 | 0.00350 |  | **B*44** | 0.02051 | 0.01983 |  | **DQB1*01** | 0.00149 | 0.00078 |
| A*68 | 0.09732 | 0.09487 |  | **B*45** | 0.00746 | 0.00972 |  | **DQB1*02** | 0.34093 | 0.34387 |
| A*69 | 0.00261 | 0.00311 |  | **B*47** | 0.00336 | 0.00272 |  | **DQB1*03** | 0.25952 | 0.24785 |
| A*74 | 0.00634 | 0.00505 |  | **B*49** | 0.02237 | 0.01827 |  | **DQB1*04** | 0.01344 | 0.01835 |
|  |  |  |  | **B*50** | 0.16480 | 0.16485 |  | **DQB1*05** | 0.13443 | 0.13271 |
| C*01 | 0.01715 | 0.02605 |  | **B*51** | 0.20768 | 0.18818 |  | **DQB1*06** | 0.24832 | 0.25449 |
| C*02 | 0.02796 | 0.02722 |  | **B*52** | 0.02088 | 0.02216 |  | **DQB1*07** | 0.00075 | 0.00078 |
| C*03 | 0.03691 | 0.04160 |  | **B*53** | 0.04400 | 0.03033 |  | **DQB1*11** | 0.00037 | 0.00000 |
| C*04 | 0.09433 | 0.10459 |  | **B*55** | 0.00522 | 0.00505 |  | **DQB1*13** | 0.00037 | 0.00039 |
| C*05 | 0.01044 | 0.01322 |  | **B*56** | 0.00000 | 0.00039 |  | **DQB1*15** | 0.00037 | 0.00078 |
| C*06 | 0.19836 | 0.19596 |  | **B*57** | 0.00858 | 0.01050 |  |  |  |  |
| C*07 | 0.25019 | 0.24961 |  | **B*58** | 0.02722 | 0.02994 |  |  |  |  |
| C*08 | 0.01902 | 0.01594 |  | **B*59** | 0.00000 | 0.00039 |  |  |  |  |
| C*11 | 0.00037 | 0.00000 |  | **B*67** | 0.00075 | 0.00000 |  |  |  |  |
| C*12 | 0.06935 | 0.06843 |  | **B*73** | 0.01007 | 0.00700 |  |  |  |  |
| C*14 | 0.01976 | 0.02255 |  | **B*81** | 0.00112 | 0.00156 |  |  |  |  |
| C*15 | 0.16182 | 0.13841 |  | **B*82** | 0.00000 | 0.00117 |  |  |  |  |
| C*16 | 0.05593 | 0.05132 |  |  |  |  |  |  |  |  |
| C*17 | 0.03579 | 0.04199 |  |  |  |  |  |  |  |  |
| C*18 | 0.00261 | 0.00272 |  |  |  |  |  |  |  |  |
| C*51 | 0.00000 | 0.00039 |  |  |  |  |  |  |  |  |

**Table 2 supplementary.** Observed and expected heterozygosity and Hardy Weinberg equilibrium significance for HLA-A, -B, -C, - DRB1 and DQB1 loci in Saudi population.

| **Loci** | **# Genotype** | **Observed**  **Heterozygosity** | **Expected Heterozygosity** | **p-value** |
| --- | --- | --- | --- | --- |
| **HLA-A** | 2773 | 0.82402 | 0.87440 | 0.00000 |
| **HLA-B** | 2773 | 0.84493 | 0.90342 | 0.00000 |
| **HLA-C** | 2773 | 0.79661 | 0.85411 | 0.00000 |
| **HLA-DRB1** | 2773 | 0.80779 | 0.86624 | 0.00000 |
| **HLA-DQB1** | 2621 | 0.66768 | 0.73724 | 0.00000 |

**Table 3 Supplementary.** Comparison of haplotype homozygosity in the study cohort, Japanese population, NMDP registry and East African populations.

|  | **Study**  **cohort** | **Japanese**  **population** | **East African** |
| --- | --- | --- | --- |
| **Haplotype homozygosity** | 4.8% | 1% | 0.96% |
